# Patient-Initiated Follow-Up in Ovarian Cancer

**DOI:** 10.3390/curroncol30040276

**Published:** 2023-03-26

**Authors:** Hiu Mei Luk, Siew Fei Ngu, Lesley S. K. Lau, Ka Yu Tse, Mandy M. Y. Chu, Shuk Tak Kwok, Hextan Y. S. Ngan, Karen K. L. Chan

**Affiliations:** Department of Obstetrics and Gynaecology, The University of Hong Kong, Queen Mary Hospital, Hong Kong SAR, China

**Keywords:** ovarian cancer, patient-initiated follow-up, fear of cancer recurrence, supportive care needs, cancer survivorship

## Abstract

This study aimed to assess the feasibility of patient-initiated follow-up (PIFU) in combination with regular tumour marker monitoring as an alternative to conventional hospital follow-up for ovarian cancer survivors. Women who had recently completed treatment for ovarian cancer and had a raised pre-treatment tumour marker were recruited. Participants were allocated to PIFU (intervention group) or conventional hospital follow-up (control group) according to their own preference. Both groups had regular tumour marker monitoring. The change in fear of cancer recurrence (FCR) score as measured by the FCR inventory, and the supportive care need (SCN) scores as measured by the SCN survey at baseline and at 6 months between PIFU and hospital follow-up were compared. Out of 64 participants, 37 (58%) opted for hospital follow-up and 27 (42%) opted for PIFU. During the 6-month study period, there was no significant difference in the change of FCR between the two groups (*p* = 0.35). There was a significant decrease in the sexuality unmet needs score in the intervention group from baseline to 6-month FU (mean difference −8.7, 95% confidence interval −16.1 to −1.4, *p* = 0.02). PIFU with tumour marker monitoring is a feasible follow-up approach in ovarian cancer survivorship care. FCR and SCN were comparable between PIFU and conventional hospital follow-up.

## 1. Introduction

Ovarian cancer is one of the commonest gynaecological cancers and has a significant worldwide effect in terms of incidence and mortality [1]. Primary treatment usually includes surgery and chemotherapy. Although recurrence is common, especially in advanced disease, the majority of patients achieve a period of remission. Currently, after completion of primary treatment, patients receive long-term regular follow-up (FU) at the hospital. Commonly, they attend the clinics every 2–4 months in the first 2 years, then every 3–6 months for the next 3 years, then yearly afterwards up to 10 years or beyond [2]. The intended purposes of these appointments are to detect recurrence, give reassurance and address any symptoms.

Whether regular FU can detect recurrences earlier and hence achieve better survival is questionable. The majority of studies have concluded that regular FU in gynaecological cancer does not significantly affect survival [3,4,5]. Moreover, studies have reported that many survivors felt their doctors were unhelpful in addressing their psychological needs once active treatment was completed and often experience anxiety prior to their hospital appointments for cancer FU [6]. A forthcoming appointment may prompt memories of cancer diagnosis, raising concerns on their health condition, leading to fear of cancer recurrence (FCR) [7]. Cancer survivors commonly experience FCR which is related to psychological distress and impaired functioning [8]. This in turn may affect their quality of life and increase health care use. Thus, balancing post-treatment surveillance with a woman’s wish to revert to normal life is paramount. Psychological interventions for FCR have been shown to be effective and could improve supportive cancer care [9]. Meanwhile, the intensive FU in the hospital add a burden to the already overloaded health system. Consequently, alternative provision of FU in the survivorship care including primary care FU, nurse-led FU, telephone-based FU and patient-initiated FU (PIFU) have been used.

PIFU allows patients to access hospital FU appointments on an “as required” basis instead of being given regular scheduled hospital appointments. Patients are educated on possible red flag symptoms and are offered direct access to specialist FU when they have any symptoms or concerns. This on-demand approach can reduce the number of unnecessary visits and possible diagnostic delay as some patients may wait for their next scheduled appointment even if they are experiencing symptoms and may provide better patient satisfaction. A meta-analysis including six studies (three on rheumatological disease, two on inflammatory bowel disease and one on breast cancer) reported that PIFU led to fewer overall outpatient appointments while sustaining comparable, if not better, clinical outcomes, patient satisfaction and quality of life [10].

In gynaecological malignancies, PIFU has been explored in the survivorship care of endometrial cancer. A randomized controlled trial (RCT) comparing PIFU with traditional hospital-based FU in early-stage endometrial cancer found that PIFU reduces health care use, but fear of recurrence decreased significantly less in PIFU [11]. Nonetheless, PIFU deserves to be fully explored for gynaecological cancer survivors. Most studies on alternative FU arrangements in gynaecological cancers have focused on endometrial cancer because of its low risk of recurrence. To date, no RCT has been reported on PIFU in ovarian cancer survivors. Ovarian cancer, unlike endometrial cancer, has a higher risk of recurrence but there is commonly a tumour marker (CA125) that is clinically useful for disease monitoring. A combination of PIFU with routine CA125 measurement may be an option that could reduce the number of hospital appointments while maintaining or improving patient reassurance, as most ovarian cancer recurrence is detected by tumour markers or symptoms [12,13].

Generally, it is a common practice to inform the patients of their test results at the next FU appointment, usually a few months later, unless the test is abnormal and warrants immediate action, then the patients would be called back earlier. A previous local study showed that 47% of cancer patients perceived moderate to high unmet needs regarding being informed about their test results in a timely manner [14], suggesting that the current clinical practice may not fulfil patients’ perceived needs. Thus, the cancer survivors may feel more reassured if they are informed of their tumour marker results as soon as it is available, usually within one week, without having to wait until the next clinic appointment.

This pilot study aimed to assess the feasibility of PIFU in combination with regular tumour marker monitoring as an alternative to standard hospital-based FU for ovarian cancer survivors. FCR and unmet supportive care needs (SCN) between PIFU and standard hospital-based FU were compared. In addition to direct or on-demand access to hospital appointments, we proposed to inform the patients of their tumour marker results as soon as they were available by short messages via phone applications such as WhatsApp which is now widely used in our local community across all age groups.

## 2. Materials and Methods

### 2.1. Trial Design

In a tertiary referral centre for gynaecological malignancies, we recruited women in a pilot study and compared the change in FCR and unmet SCN scores at baseline and at 6 months between PIFU and conventional hospital-based FU in the survivorship care of ovarian cancer. We hypothesized that PIFU with early notification of tumour marker results could achieve a comparable patient reassurance (as measured by FCR score) and satisfaction (as measured by unmet SCN scores) compared with conventional hospital-based FU, while reducing the number of FU appointments. This study was approved by the Institutional Review Board of the University of Kong Hong/Hospital Authority Hong Kong West Cluster and conducted in accordance with the principles of the Declaration of Helsinki.

### 2.2. Study Population

Women aged ≥18 years who had recently completed treatment (within 24 months) for ovarian cancer and were having 3-monthly clinic FUs as well as met the inclusion criteria were recruited. Those with ovarian carcinoma or borderline ovarian tumour of any stage and histological type, had a raised pre-treatment tumour marker (CA125, CA19.9 or CEA) and were able to understand the Chinese versions of the questionnaires were eligible. Women who had other active cancer within 2 years, other co-existing medical conditions rendering tumour marker monitoring unreliable, on-going clinical issues that warranted medical attention and those who were unable to attend regular FU appointments as scheduled were excluded. All eligible women were provided with study information by a designated research assistant and those who agreed to participate in the study provided written informed consent.

### 2.3. Study Interventions

Women who agreed to participate in the study were allocated to PIFU (intervention group) or conventional gynaecological oncologist-led clinic FU (control group) according to their own preference. For the control group, women attended hospital-based outpatient clinic FU according to our standard schedule (3-monthly FUs in the first 2 years, then 6-monthly FUs in the next 3 years, and then yearly afterward) for symptom review and physical examination. They also had tumour marker (CA125 +/− CA19.9 +/− CEA) monitoring according to the routine care (for stage I ovarian cancer: 3-monthly; for stage II-IV: 6-weekly in the first year; then 3-monthly in the second year) and were informed of their results at their next FU appointment 3 months later, unless the test was abnormal and warranted immediate action, then the women would be called back earlier. Patient-reported outcomes were not included in our routine cancer FU. For the PIFU group, women had no scheduled FU appointment until they returned to the standard FU schedule at the end of the 6-month study period, but they had regular tumour marker monitoring according to the routine care mentioned above. In addition, they were given information on the symptoms of recurrence and a direct telephone line to contact our research assistant (who is experienced in clinical trials on gynaecological cancers and has basic knowledge in survivorship care) if they had any concerns. The research assistant would then contact the gynaecological oncologist and arrange a hospital appointment for them within 1 to 2 weeks of reviewing the concerns involved. Furthermore, women in the PIFU group were informed of their tumour marker results and any further instructions by short telephone message (by WhatsApp) as soon as the results were available, usually within 1 week. At the end of the 6-month study period, participants in the PIFU group returned to the standard FU schedule.

All recruited women completed the fear of cancer recurrence inventory—short form (FCRI-SF) and the supportive care needs survey—short form (SCNS-SF34) questionnaires at baseline (at recruitment) and 6 months later. FCRI-SF is a 9-item instrument assessing the presence, frequency, intensity and duration of thoughts related to FCR. Each item is rated on a 5-point Likert scale (0 = never; 4 = a great deal or all the time). Total scores range from 0 to 36, with higher scores indicating greater FCR. Scores of 13 to 21 and ≥22 represent subclinical and clinical cut-offs, respectively. The FCRI-SF has demonstrated strong internal consistency (Cronbach a = 0.89), convergent validity and test–re-test reliability [15]. The Chinese version has been validated and used in published local studies [16,17]. The SCNS-SF34 is a 34-item instrument that assesses levels of unmet needs in cancer patients across five domains: psychological (10 items), health systems and information (11 items), physical and daily living (5 items), patient care and support (5 items) and sexuality (3 items) [18]. Patients reported the magnitude of each specified need over the past month on a 5-point Likert scale (1 = no need, not applicable; 2 = no need, satisfied; 3 = low need; 4 = moderate need; 5 = high need). The Chinese SCNS-SF34 has demonstrated good internal reliability (Cronbach’s a > 0.70) and construct validity within the Hong Kong population [19].

### 2.4. Study Outcome 

The primary outcome was the FCR as measured by the FCRI after 6 months of FU. The secondary outcomes included the supportive care need (SCN) scores as measured by the SCNS after 6 months of FU, time to detection of recurrence (if patients recurred during the study period), total number of hospital visits (for ovarian cancer FU only) and number of phone contacts by patients.

### 2.5. Sample Size and Statistical Analysis

This is a pilot study looking at the feasibility for a larger RCT between the intervention and control arms. Since psychosocial behavioural interventions usually have a small effect size, a small effect size was used in the calculation of sample size. Using the stepped rule of thumb for pilot study sample size, assuming a small effect size (0.1 ≤ standardized difference < 0.3) and 90% powered main trial, a total of 50 participants was needed. The differences in the background demographics between the two groups were compared with the Mann–Whitney U test, chi-square test or Fisher exact test. Mean scores of the FCR and SCN were compared using Student’s *t*-tests. All statistical analysis was conducted using SPSS version 26.0 (Armonk, NY, USA, IBM Corp). A *p* value of < 0.05 was considered statistically significant.

## 3. Results

Between 1 April 2020 and 27 January 2021, a total of 72 women with ovarian cancer were assessed for eligibility of which 64 women were recruited and 8 declined to participate. Of those who agreed to participate, 37 (58%) women opted for conventional gynaecological oncologist-led clinic FU (control group) and 27 (42%) women opted for PIFU (intervention group). The background demographics of the participants are presented in Table 1. There was no significant difference between the two groups in terms of age, disease stage, time from end of active cancer treatment, number of recurrences and time to detection of recurrence after joining the study. The mean age of women in the intervention group was 48 years, which was younger than the control group with a mean age of 54 years. During the study period, five women (three in the control group and two in the intervention group) were diagnosed with disease recurrence, all of which were detected following a raised CA125 and then confirmed with imaging. The extent of disease at detection of recurrence between the two groups appeared to be similar, although the number of recurrences were too small for a meaningful comparison. All five women had peritoneal recurrence while one woman in each group also had nodal recurrence. There was no significant difference in the time to detection of recurrence between the two groups.

A total of 64 FCRI-SF and SCNS-SF34 questionnaires were available for analysis at baseline (Table 2). At 6-month FU, 54 (84%) questionnaires were available for analysis (Table 2). In total, 10 women did not complete the questionnaires at 6 months due to disease recurrence (*n* = 5), early termination (*n* = 2) and refusal to complete (*n* = 3).

At baseline, women in the control group had a mean FCR total score of 14.6 (standard deviation (SD) 7.0), and this score remained similar with an improvement of 0.5 at 6-month FU. In the intervention group, the baseline score was 13.7 (SD 7.3). At 6-month FU, this score had deteriorated slightly by 1.0. During the 6-month study period, there was no significant difference in the change of FCR between the two groups (*p* = 0.35) (Table 2).

In both the control and intervention group, the scores of unmet SCN in the health system and information domain improved from baseline to 6-month FU. In the intervention group, there were also improvements in the physical and daily living unmet need scores, and patient care and support unmet need scores from baseline to 6-month FU. Additionally, there was a significant decrease in the sexuality unmet needs score in the intervention group from baseline to 6-month FU (mean difference −8.7, 95% confidence interval (CI) −16.1 to −1.4, *p* = 0.02) (Table 2).

In the multivariate analysis, the FCR and SCN scores at 6-month FU were significantly correlated with the scores at baseline (*p* < 0.01). Younger age was significantly correlated with more psychological unmet needs (*p* = 0.03) and sexuality unmet needs (*p* = 0.03).

There was no significant difference between the two groups in the number of ovarian cancer-related hospital FU visits (*p* = 0.85). However, during the study period, the median number of FUs in the control group and the intervention group was two (at 3 months and 6 months) and one (at 6 months), respectively.

Overall, 11 (41%) women in the intervention group contacted the research assistant at least once during the study period, of which 4 (15%) had an earlier patient-initiated clinic FU arranged (1 for abdominal pain, limbs pain and abdominal distension; 1 for constipation; 1 for peripheral neuropathy; and 1 was worried about an ultrasound scan finding). The remaining seven women had their concerns or queries resolved with telephone messages. Some of the queries received were unrelated to ovarian cancer, such as enquiries regarding blood donation, flu vaccination and hospital appointments in the other specialties.

After the end of the study period, participants in the intervention group returned to routine FU according to local practice. Of the 23 women in the PIFU group who were asked about their preferred FU method having experienced both PIFU and hospital-based FU, 12 (52%) preferred PIFU, 3 (13%) preferred hospital-based FU, 1 (4%) expressed that it would depend on the FU interval, and 7 (30%) did not give a response.

## 4. Discussion

This was a pilot study to explore the feasibility of PIFU in the survivorship care of ovarian cancer. Overall, 42% of participants opted for PIFU as supposed to the conventional hospital FU. Furthermore, 52% of participants who had experienced both PIFU and hospital FU preferred PIFU for their survivorship care. These findings are significant in our locality considering that patients are more familiar with the traditional hospital FU. Without knowing if our patients are willing to accept PIFU, we could not implement this pathway in our survivorship care or plan for a larger study to evaluate its effect on the cancer survivorship care. In view of these findings, we believe that the utilization and implementation of PIFUs in ovarian cancer survivorship care is feasible in our community.

The differences in FCR scores between PIFU and hospital-based FU were comparable (*p* = 0.35), with changes in FCR scores from baseline to 6-months’ FU of −0.5 versus 1.0 for hospital-based FU and PIFU groups, respectively. In a recent systemic review, FCR was reported in three studies (one endometrial, one breast, one prostate), and all used different tools for assessment [20]. Of these, only one RCT on early-stage endometrial cancer found significant differences in FCR between PIFU and hospital-based FU, where FCR scores decreased significantly more in the hospital-based FU from baseline to 10 months of FU (difference −5.9, 95% CI −10.9 to −0.9, *p* = 0.02) [11]. Nonetheless, the proportion of women with clinical FCR was comparable at 10 months regardless of the type of FU, with around 20% of women affected by FCR at 10 months post-treatment. Our findings on FCR were consistent with other studies on breast and prostate cancer [21,22]. It may be because there was no provision of routine FU for the women in the PIFU group and there was no tumour marker monitoring in the endometrial cancer study, which may contribute to increased anxiety and fear.

In this pilot study, the number of cancer recurrences was similar in the two groups, and all were detected by raised CA125 levels. Our previous RCT on nurse-led telephone FU versus conventional doctor-led FU at the specialist clinic showed that at a median FU of 23 months, out of 170 ovarian cancer patients, there were 9 recurrences [23]. Of these, five recurrences were detected by raised CA125 alone, two presented to the other doctors in the private clinic before their routine FU appointment and two attended our unit with symptoms before their routine FU. This suggested that routine appointments for physical examination have very little value in the early detection of ovarian cancer recurrences. Our findings are consistent with other studies that reported a limited role of regular physical examination for detection of ovarian cancer recurrence, as most were detected by tumour markers or symptoms [12,13,24]. Indeed this finding was consistent with published data suggesting that for many cancers, recurrences are not commonly detected in asymptomatic patients at FU consultations and most recurrences are reported as interval events [25]. A recent systemic review reported that although the included studies were insufficiently powered to determine a difference between PIFU and conventional hospital FU in terms of recurrence rates, results tended to show no significant differences [20].

Recently, there has been an increased utilization of PIFUs in the cancer survivorship care, especially during the COVID-19 pandemic due to the reduction in outpatient hospital appointments. In fact, recommendations and guidance on the implementation of PIFUs in gynaecological cancers have been published [26]. PIFUs have been explored in other cancers, although there have only been a few RCTs published. A recent systemic review found that of the eight studies included, six were RCTs (one endometrial, one colorectal, four breast) [20]. Of these, only one study with 106 colorectal cancer patients reported overall survival as the primary outcome. Although this study was not adequately powered to detect a difference in survival, it demonstrated no significant differences between PIFU and hospital-based FU [27]. Generally, it was found that patient satisfaction with hospital-based FU and PIFU were similar. Patients in the hospital-based FU tended to be more reassured by face-to-face visits, while patients in PIFU tended to find this mode of FU more convenient. Furthermore, PIFU has not been found to have a negative impact on the phycological morbidity or quality of life [20].

In a previous local study on supportive care needs for cancer patients, the most common unmet need was “Having one member of hospital staff with whom you can talk to about all aspects of your condition, treatment and follow up” [14]. Despite identification of this as an important unmet need, so far, it is not possible to provide such personalized service in most of our local public hospital system. Of the women who agreed to participate in this pilot study, 42% opted for PIFUs and these women tended to be younger. In the multivariate analysis, younger age was significantly correlated with more psychological and sexuality unmet needs. In the PIFU group, apart from less frequent hospital visit, women could contact a dedicated research assistant directly if they had any concern or to make an appointment if needed. Furthermore, these women were informed of their tumour marker results as soon as they were available (usually within 1 week) by WhatsApp text messaging instead of having to wait until the next FU appointment 3 months later. These arrangements may be perceived as more flexible and women may feel less anxious after knowing their tumour marker results. Consequently, these may lead to the improvement in the unmet supportive care needs, particularly in the health system and information, physical and daily living, patient care and support, and sexuality domain in the PIFU group.

In the present study, most women who chose routine hospital FU have already experienced the hospital appointments every 3–4 months for 1–2 years, and thus may have felt more reassured and preferred to continue this mode of FU. Indeed, a recent survey on the preferences of endometrial cancer patients on FU showed that patients tended to prefer a FU method that was familiar to them [28]. In contrast, most women who opted for PIFUs had just completed active cancer treatment and were more naive to the different methods of FU. Consequently, when planning for subsequent studies, patients who have just completed their active cancer treatment and are new to the survivorship care should be the target for the study population. As this study was conducted during the COVID-19 pandemic, participants may have preferred less frequent appointments in PIFU so that they can avoid frequent hospital visits and being exposed to the COVID-19 infection. Therefore, further studies are needed to support the implementation of PIFUs on a longer-term basis.

A previous study including 228 women with endometrial cancer reported that around 20% of women enrolled to PIFU contacted a clinical nurse specialist at least once during the study period [29]. In our study, 41% of women in the PIFU group contacted the research assistant at least once during the study period. Although some contacts were related to ovarian cancer, there were some unrelated enquiries encountered. Hence, clear procedures and reasons for making contact with the specialist services will be important for the effective implementation of PIFU.

Apart from PIFU, other FU approaches in ovarian cancers have been studied [30,31]. An RCT on 112 ovarian cancer patients randomized to an individualized nurse-led FU and standard FU concluded that individualized FU was superior to standard FU in terms of quality of life and patient satisfaction [31]. Our previous study comparing nurse-led telephone FU and conventional hospital FU also reported similar findings [23]. However, telephone FU was noted to be more costly to the National Health Service in the United Kingdom as it consumed more time and required experienced senior nurses [32,33]. In our previous study, we also identified problems with nurse-led telephone FU, in that it was very time consuming for the nurse to conduct these telephone FUs and it posed a pressure on the nurse to deal with the variety of related or unrelated issues that the patients raised during the telephone conversation [23]. As such, this may not be a sustainable option in our community and thus a justifiable reason for conducting the present study to evaluate PIFU in the survivorship care of ovarian cancer.

To our knowledge, this pilot study is the first study to examine the use of PIFU in ovarian cancer survivorship care. Although the sample size was small, our study showed that some women were willing to choose PIFU and preferred this method of FU. Furthermore, our study compared outcomes in a relatively short period of time (over 6 months), when significant events or changes may not have occurred yet. Thus, further randomized control trials with larger sample size and longer FU period are required. Eventually, PIFU and tumour marker monitoring with or without a longer interval of routine hospital-based FU may be considered in the survivorship care of ovarian cancer.

## 5. Conclusions

PIFU with tumour marker monitoring is a feasible follow-up approach in ovarian cancer survivorship care. FCR and SCN were comparable between PIFU and conventional hospital follow-up. It may reduce unnecessary FU and hence increase the resources for patients who truly need the medical service. Ultimately, implementing PIFU in the cancer survivorship program may improve patient satisfaction and quality of life, and hopefully can enhance clinical outcomes in the long run.

## Figures and Tables

**Table 1:** Demographics of participants.

	Patient-Initiated Follow-Up *N* = 27, *n* (%)	Hospital-Based Follow-Up *N* = 37, *n* (%)	*p* Value
Mean age (years) (SD)	47.7 (13.6)	53.8 (12.7)	0.066
FIGO Stage			0.125
Borderline ovarian tumour			
I	5 (19)	3 (8)	
III	3 (11)	0 (0)	
Ovarian carcinoma			
I	6 (22)	16 (43)	
II	4 (15)	4 (11)	
III	4 (15)	9 (24)	
IV	5 (19)	5 (14)	
Time from end of active cancer treatment			0.110
Newly follow-up after cancer treatment	14 (52)	11 (30)	
<1 year	7 (26)	9 (24)	
1–2 years	6 (22)	17 (46)	
Number of recurrences	2 (7)	3 (8)	0.649
Mean time to detection of recurrence (months) (95% confidence interval)	6.7 (6.4–7.1)	8.8 (8.1–9.4)	0.745

SD, standard deviation. Data are given as *n* (%) unless otherwise stated.

**Table 2:** Mean summary scores and standard deviation for the fear of cancer recurrence inventory—short form and supportive care needs survey—short form for both groups at baseline and 6-month follow-up, and differences in scores between baseline and 6 months.

	Baseline *N* = 64	6 Months *N* = 54	Difference	*p* Value
Total FCRI-SF score				0.350
Intervention	13.7 (7.3)	14.1 (6.9)	1.0 (4.8)	
Control	14.6 (7.0)	14.0 (7.5)	−0.5 (4.7)	
% with clinical FCR				0.683
Intervention	11.1	13.6		
Control	16.2	12.5		
SCNS-SF domains				
Psychological				0.798
Intervention	25.4 (24.7)	25.3 (21.3)	3.4 (11.8)	
Control	28.9 (23.3)	32.3 (27.8)	2.1 (19.8)	
Health System and Information				0.628
Intervention	39.2 (29.8)	36.1 (23.6)	−3.2 (21.2)	
Control	42.3 (24.0)	36.4 (29.3)	−4.4 (25.6)	
Physical and Daily Living				0.101
Intervention	26.7 (24.5)	16.4 (13.8)	−7.0 (17.9)	
Control	22.5 (25.1)	27.8 (23.2)	2.8 (17.1)	
Patient Care and Support				0.756
Intervention	27.6 (24.7)	23.9 (23.3)	−1.4 (22.1)	
Control	29.1 (21.1)	31.9 (30.4)	3.3 (21.8)	
Sexuality				0.003
Intervention	17.6 (22.9)	7.2 (15.3)	−8.7 (16.6) *	
Control	14.4 (23.6)	17.4 (26.9)	4.2 (15.7)	

FCRI-SF, fear of cancer recurrence inventory—short form; FCR, fear of cancer recurrence; SCNS-SF, supportive care needs survey—short form. Differences in scores between baseline and 6 months included only women (*N* = 54) who completed questionnaires at baseline and 6 months. Standard deviations are given in parentheses. *p* values are comparisons in the difference in scores between 2 groups using Mann–Whitney U test. * *p* < 0.05 for the difference between baseline and 6 months in the intervention group.

## Data Availability

The data presented in this study are available on request from the corresponding author. The data are not publicly available due to ethical reasons.

## References

[1] The Global Cancer Observatory Cancer Today. https://gco.iarc.fr/today/data/factsheets/cancers/23-Cervix-uteri-fact-sheet.pdf.

[2] National Comprehensive Cancer Network Clinical Practice Guidelines in Oncology: Cervical Cancer. https://www.nccn.org/guidelines/guidelines-detail?category=1&id=1426.

[3] Lajer H., Jensen M.B., Kilsmark J., Albæk J., Svane D., Mirza M.R., Geertsen P.F., Reerman D., Hansen K., Milter M.C. (2010). The value of gynecologic cancer follow-up: Evidence-based ignorance?. Int. J. Gynecol. Cancer.

[4] Salani R., Backes F.J., Fung M.F.K., Holschneider C.H., Parker L.P., Bristow R.E., Goff B.A. (2011). Posttreatment surveillance and diagnosis of recurrence in women with gynecologic malignancies: Society of Gynecologic Oncologists recommendations. Am. J. Obstet. Gynecol..

[5] Jeppesen M.M., Mogensen O., Hansen D.G., Bergholdt S.H., Jensen P.T. (2019). How Do We Follow Up Patients With Endometrial Cancer?. Curr. Oncol. Rep..

[6] Lewis R.A., Neal R.D., Hendry M., France B., Williams N.H., Russell D., Hughes D.A., Russell I., Stuart N.S., Weller D. (2009). Patients’ and healthcare professionals’ views of cancer follow-up: Systematic review. Br. J. Gen. Pract..

[7] Bradley E., Pitts M., Redman C., Calvert E. (1999). The experience of long-term hospital follow-up for women who have suffered early stage gynecological cancer: A qualitative interview study. Int. J. Gynecol. Cancer.

[8] Luigjes-Huizer Y.L., Tauber N.M., Humphris G., Kasparian N.A., Lam W.W.T., Lebel S., Simard S., Smith A.B., Zachariae R., Afiyanti Y. (2022). What is the prevalence of fear of cancer recurrence in cancer survivors and patients? A systematic review and individual participant data meta-analysis. Psycho-Oncology.

[9] Tauber N.M., O’Toole M.S., Dinkel A., Galica J., Humphris G., Lebel S., Maheu C., Ozakinci G., Prins J., Sharpe L. (2019). Effect of Psychological Intervention on Fear of Cancer Recurrence: A Systematic Review and Meta-Analysis. J. Clin. Oncol..

[10] Taneja A., Su’A B., Hill A. (2014). Efficacy of patient-initiated follow-up clinics in secondary care: A systematic review. Intern. Med. J..

[11] Jeppesen M.M., Jensen P.T., Hansen D.G., Christensen R.D., Mogensen O. (2018). Patient-initiated follow up affects fear of recurrence and healthcare use: A randomised trial in early-stage endometrial cancer. BJOG Int. J. Obstet. Gynaecol..

[12] Janke M.J., Santiago S., Straubhar A.M., Uppal S. (2022). The utility of physical examination in ovarian cancer recurrence detection: A retrospective analysis informing virtual surveillance care. Int. J. Gynecol. Cancer.

[13] Feinberg J., Carthew K., Webster E., Chang K., McNeil N., Chi D.S., Roche K.L., Gardner G., Zivanovic O., Sonoda Y. (2022). Ovarian cancer recurrence detection may not require in-person physical examination: An MSK team ovary study. Int. J. Gynecol. Cancer.

[14] Li W.W.Y., Lam W.W.T., Au A.H.Y., Ye M., Law W.L., Poon J., Kwong A., Suen D., Tsang J., Girgis A. (2013). Interpreting differences in patterns of supportive care needs between patients with breast cancer and patients with colorectal cancer. Psycho-Oncology.

[15] Simard S., Savard J. (2009). Fear of Cancer Recurrence Inventory: Development and initial validation of a multidimensional measure of fear of cancer recurrence. Support. Care Cancer.

[16] Ng D.W.L., Kwong A., Suen D., Chan M., Or A., Ng S.S., Foo C.C., Fielding B.F., Lam W.W. (2019). Fear of cancer recurrence among Chinese cancer survivors: Prevalence and associations with metacognition and neuroticism. Psycho-Oncology.

[17] Ng D.W.L., Foo C., Ng S.S.M., Kwong A., Suen D., Chan M., Or A., Chun O.K., Fielding B.F.S., Lam W.W.T. (2020). The role of metacognition and its indirect effect through cognitive attentional syndrome on fear of cancer recurrence trajectories: A longitudinal study. Psycho Oncol..

[18] Boyes A., Girgis A., Lecathelinais C. (2009). Brief assessment of adult cancer patients’ perceived needs: Development and validation of the 34-item Supportive Care Needs Survey (SCNS-SF34). J. Evaluation Clin. Pract..

[19] Au A., Lam W.W.T., Kwong A., Suen D., Tsang J., Yeo W., Suen J., Ho W.M., Yau T.K., Soong I. (2011). Validation of the Chinese version of the Short-form Supportive Care Needs Survey Questionnaire (SCNS-SF34-C). Psycho Oncol..

[20] Newton C., Beaver K., Clegg A. (2022). Patient initiated follow-up in cancer patients: A systematic review. Front. Oncol..

[21] Sheppard C., Higgins B., Wise M., Yiangou C., Dubois D., Kilburn S. (2009). Breast cancer follow up: A randomised controlled trial comparing point of need access versus routine 6-monthly clinical review. Eur. J. Oncol. Nurs..

[22] Frankland J., Brodie H., Cooke D., Foster C., Foster R., Gage H., Jordan J., Mesa-Eguiagaray I., Pickering R., Richardson A. (2019). Follow-up care after treatment for prostate cancer: Evaluation of a supported self-management and remote surveillance programme. BMC Cancer.

[23] Ngu S., Wei N., Li J., Chu M.M.Y., Tse K.Y., Ngan H.Y.S., Chan K.K.L. (2020). Nurse-led follow-up in survivorship care of gynaecological malignancies—A randomised controlled trial. Eur. J. Cancer Care.

[24] Chan K., Tam K., Tse K., Ngan H. (2008). The role of regular physical examination in the detection of ovarian cancer recurrence. Gynecol. Oncol..

[25] Jefford M., Rowland J., Grunfeld E., Richards M., Maher J., Glaser A. (2013). Implementing improved post-treatment care for cancer survivors in England, with reflections from Australia, Canada and the USA. Br. J. Cancer.

[26] Newton C., Nordin A., Rolland P., Ind T., Larsen-Disney P., Martin-Hirsch P., Beaver K., Bolton H., Peevor R., Fernandes A. (2020). British Gynaecological Cancer Society recommendations and guidance on patient-initiated follow-up (PIFU). Int. J. Gynecol. Cancer.

[27] Ohlsson B., Breland U., Ekberg H., Graffner H., Tranberg K.-G. (1995). Follow-up after curative surgery for colorectal carcinoma. Randomized comparison with no follow-up. Dis. Colon Rectum.

[28] Beaver K., Williamson S., Sutton C.J., Gardner A., Martin-Hirsch P. (2020). Endometrial cancer patients’ preferences for follow-up after treatment: A cross-sectional survey. Eur. J. Oncol. Nurs..

[29] Kumarakulasingam P., McDermott H., Patel N., Boutler L., Tincello D.G., Peel D., Moss E.L. (2019). Acceptability and utilisation of patient-initiated follow-up for endometrial cancer amongst women from diverse ethnic and social backgrounds: A mixed methods study. Eur. J. Cancer Care.

[30] Cox A., Bull E., Cockle-Hearne J., Knibb W., Potter C., Faithfull S. (2008). Nurse led telephone follow up in ovarian cancer: A psychosocial perspective. Eur. J. Oncol. Nurs..

[31] Lanceley A., Berzuini C., Burnell M., Gessler S., Morris S., Ryan A., Ledermann J.A., Jacobs I. (2017). Ovarian Cancer Follow-up: A Preliminary Comparison of 2 Approaches. Int. J. Gynecol. Cancer.

[32] Beaver K., Hollingworth W., McDonald R., Dunn G., Tysver-Robinson D., Thomson L., Hindley A.C., Susnerwala S.S., Luker K. (2009). Economic evaluation of a randomized clinical trial of hospital *versus* telephone follow-up after treatment for breast cancer. Br. J. Surg..

[33] Dixon P., Beaver K., Williamson S., Sutton C., Martin-Hirsch P., Hollingworth W. (2018). Cost-Consequence Analysis Alongside a Randomised Controlled Trial of Hospital Versus Telephone Follow-Up after Treatment for Endometrial Cancer. Appl. Health Econ. Health Policy.

